# Graphene Oxide-Assisted Promotion of Plant Growth and Stability

**DOI:** 10.3390/nano10040758

**Published:** 2020-04-15

**Authors:** Sunho Park, Kyoung Soon Choi, Sujin Kim, Yonghyun Gwon, Jangho Kim

**Affiliations:** 1 Department of Rural and Biosystems Engineering, Chonnam National University, Gwangju 61186, Korea; preference9330@gmail.com (S.P.); kimsujin4172@gmail.com (S.K.); gyhjhj0716@gmail.com (Y.G.); 2 National Research Facilities & Equipment center (NFEC), Korea Basic Science Institute (KBSI), 169-148, Gwahak-ro, Yuseong-gu, Daejeon 34133, Korea; kschoi@kbsi.re.kr

**Keywords:** plant growth, nanomaterials, graphene oxide, *Arabidopsis thaliana* L., watermelon

## Abstract

The control and promotion of plant and crop growth are important challenges globally. In this study, we have developed a nanomaterial-assisted bionic strategy for accelerating plant growth. Although nanomaterials have been shown to be toxic to plants, we demonstrate herein that graphene oxide can be used as a regulator tool for enhancing plant growth and stability. Graphene oxide was added to the growth medium of *Arabidopsis thaliana* L. as well as injected into the stem of the watermelon plant. We showed that with an appropriate amount provided, graphene oxide had a positive effect on plant growth in terms of increasing the length of roots, the area of leaves, the number of leaves, and the formation of flower buds. In addition, graphene oxide affected the watermelon ripeness, increasing the perimeter and sugar content of the fruit. We believe that graphene oxide may be used as a strategy for enabling the acceleration of both plant growth and the fruit ripening process.

## 1. Introduction

Given the rapid population growth and climate change worldwide, the needs for efficient food cultivation and food security have become ever more important. Therefore, various approaches have been proposed to increase crop yields and to protect the growth of crops and vegetation under various environmental conditions, including extreme temperature changes and natural disasters [1,2,3]. Among the various approaches, those based on chemical materials have been mainly used in both crop cultivation and plant biology [4,5]. Chemical materials can provide nutrients to crops directly, and they can optimize the efficiency of the soil condition by increasing its water retention or modifying its aeration [6]. In addition, chemical materials can be easily mass produced, making their increasingly essential use in crop cultivation economically viable. However, the excessive use of chemical materials has promoted the emergence of genetic resistance in plants and crops, and the accumulation of these chemicals in soil and water can lead to serious environmental pollution. In addition, the accumulation of these chemical materials in the crops themselves can indirectly cause negative side effects to humans and animals [7]. 

As an alternative, we have devised a novel strategy using engineered nanomaterials to maximize the acceleration of plant growth and to minimize environmental pollution. Nanomaterials, which have a dimension of 1–100 nm, have been applied in various fields, including for electronic, biomedical, energy, and environmental applications, because of their outstanding physicochemical properties (e.g., large surface area and high mechanical strength) [8]. Recently, nanomaterials have been regarded as a significant breakthrough to solving the various problems in agricultural fields. For instance, Liu et al. demonstrated that carbon-based nanomaterials (i.e., carbon nanotubes (CNTs)) could penetrate the cell wall and cell membrane, and the conjugate of CNTs and DNA could also penetrate the plant cell wall [9]. In other words, there is room for engineered nanomaterials to be used as effective platforms for delivering plant growth-promoting molecules into the plant cells, while at the same time reducing environmental pollution owing to only a small amount of the nanomaterials being needed and the targeting of specific cells that possess special functions. Giraldo et al. demonstrated the transport and localization of single-walled carbon nanotubes (SWCNTs) into the chloroplast lipid bilayers through kinetic trapping, which promoted photosynthetic activity and electron transport [10]. In addition, the same group revealed that functionalized SWCNTs, which were allowed to infiltrate the plant organelles via the lamina, could act as detectors of nitroaromatics by emitting infrared fluorescence. The nitroaromatics that were transported from the roots to the leaves and accumulated in the mesophyll were recognized by the SWCNTs and the emission intensity was changed, unlike that with control SWCNTs that had an invariant reference signal [11]. 

As mentioned above, engineered nanomaterials have great potential for use in the agricultural field; however, most of the studies on nanomaterials have focused on their toxicity to plants and crops. Begum and Fugetsu reported that multi-walled carbon nanotubes (MWCNTs) of 20, 200, 1000, and 2000 mg/L were phytotoxic to red spinach, lettuce, rice, and cucumber, and the shoot and root weights were dramatically decreased with increasing nanotube concentrations [12]. Nair et al. showed that the phenotype of mung bean was regulated by media containing copper oxide nanoparticles of 20, 50, 100, 200, and 500 mg/L, and the reactive oxygen species generation in the roots increased gradually with the increase of the nanoparticle concentration [13]. In addition, nanomaterials, such as graphene, CNTs, and copper oxide nanoparticles, have been shown to be cytotoxic to various crops [14,15,16,17]. In these previous studies, a high nanomaterial concentration in the milligram per liter (mg/L) unit was mainly used, which resulted in dramatic negative changes to the phenotype and growth of plants and crops. 

In this study, we demonstrated that small concentrations of graphene oxide (GO) promoted good plant growth and stability, suggesting that this nanomaterial can render positive effects on the growth and quality of plants and crops. Our findings provide an insight into the rational design of an efficient nanomaterial-assisted cultivation system that could be used for the acceleration of both plant growth and the fruit ripening process.

## 2. Materials and Methods 

### 2.1. Fabrication of the Nanomaterials

GO was prepared by the method described by Hummers, with slight modification [18,19]. In brief, 5 g of graphite and 2.5 g of sodium nitrate (Sigma-Aldrich, St. Louis, MO, USA) were mixed with 115 mL of sulfuric acid (Sigma-Aldrich) under constant stirring in an ice bath. After 30 min, 15 g of KMnO_4_ (Sigma-Aldrich) was added slowly to the solution while keeping the temperature at less than 20 °C. The mixture was then stirred at 30 °C for 30 min and the resulting solution was diluted by the addition of 230 mL of hot water under vigorous stirring. The solution was further treated with 30% H_2_O_2_ solution (50 mL, DAEJUNG, Seoul, Korea) and 400 mL of water. The resulting mixture was washed with 20% HCl (50 mL, DAEJUNG) and H_2_O, respectively. The as-prepared GO was sonicated for 2 h before use (VS-505 sonicator; Sonics and Materials, Inc., Newtown, CT, USA). 

### 2.2. Plant Growth and Treatment

Seeds of *Arabidopsis thaliana* L. were surface sterilized with 70% ethanol and 20% sodium hypochlorite. The GO solution that had been sonicated for 2 h was blended with the nutrient medium by stirring. The Murashige and Skoog (MS) nutrient medium (DUCHEFA, Haarlem, The Netherlands) used was composed of 2.2 g of MS nutrient, 10 g of agar (DUCHEFA, Haarlem, The Netherlands), 0.5 g of 2-(*N*-morpholino) ethane sulfonic acid powder (Sigma-Aldrich, St. Louis, MO, USA), and 30 g of sucrose (Sigma-Aldrich, St. Louis, MO, USA) in 1 L of deionized water [20]. After adjusting the pH to 5.6–5.8, the MS medium was sonicated together with the GO solution, and the resulting medium (hereinafter referred to as the GO/MS medium) was sterilized in an autoclave (DH18CAT00210081; Daihan Scientific, Wonju, Gangwon, Korea). To maintain the sterilized condition, the plates were dried in a biosafety hood for one day. The plants were maintained at 4 °C for three days in darkness for stratification and then transferred to normal growth conditions with/without 100, 1000, or 10,000 μg/L of GO. The plants were grown at 24 °C under long-day conditions (24-h light) in an incubator (HB-101; Hanbaek, Korea). Each germination assay (*n* = 4) was carried out using 90–100 seeds. To determine the effects of the nanomaterial on plant seedling growth, the root length, leaf area, and number of leaves of the seedlings were measured at 24 and 30 days after growth on MS medium with/without 100, 1000, or 10,000 μg/L of GO. To measure the flowering of *A. thaliana*, each assay (*n* = 6) was carried out with 10 seeds.

### 2.3. Watermelon Growth

Watermelon seedlings were planted on same-sized Styrofoam pots, and all but three stems were removed to encourage plant growth. GO (10 μL of 10,000 μg/L) was injected into one watermelon plant through a scratch made on the stem using tweezers. The GO solution was injected once a week for one month. The perimeter of the watermelon was checked once every two days.

### 2.4. Analysis of Graphene Oxide, Arabidopsis thaliana L., and Watermelon 

Laser-Raman spectroscopy and atomic force microscopy (AFM) analyses were performed using a Complex AFM-Raman Spectroscope (NTEGRA Spectra, Shanghai, China). X-Ray photoelectron spectroscopy (XPS) and X-ray and UV photoelectron spectromicroscopy were performed using the AXIS Nova and AXIS Ultra DLD systems (AXIS Nova, KRATOS ANALYTICAL, Kyoto, Japan), respectively. The root length, leaf number, and leaf area of *A. thaliana* L. were measured using the ImageJ program (ImageJ, Bethesda, MD, USA). The leaf weight was measured on an analytical balance (ABS-N/ABJ-NM; KERN, Balingen, Germany). The sugar concentration of the watermelon was confirmed using a digital refractometer (Atago pal-1, Minato-ku, Tokyo, Japan).

## 3. Results and Discussion

### 3.1. Fabrication and Characterization of Graphene Oxide

**Figure 1:**
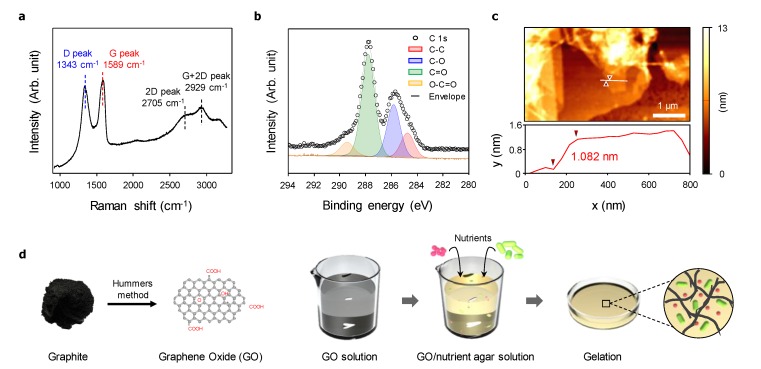
Fabrication and characteristics of graphene oxide (GO). (**a**) Raman spectroscopy, (**b**) X-Ray photoelectron spectroscopy, and (**c**) atomic force microscopy analyses of the fabricated GO. (**d**) Schematic of GO/Murashige and Skoog (MS) medium plate fabrication.

The graphite was oxidized, exfoliated, and washed to form the GO layer according to the method described by Hummers [19]. Raman spectroscopy analysis of the fabricated GO layer revealed two strong peaks at 1343 and 1589 cm^−^^1^, indicating the specific D peak (~1350 cm^−^^1^) and G peak (~1590 cm^−^^1^) of GO (Figure 1a). The G peak represents a ring-breathing mode from the sp^2^ carbon rings, and the D peak represents an in-plane vibrational mode. The shift of the G peak relative to that of graphite indicated the presence of isolated double bonds, and the increased D peak indicated that the graphite form had been defected, bearing various functional groups, including –O–, –OH, and –COOH [21]. Specifically, Figure 1b shows the C 1s XPS spectrum of GO, where the C–C (284.6 eV), C–O (285.8 eV), C=O (288.0 eV), and O–C=O (289.6 eV) components were confirmed in the C 1s peak. The atomic configurations were C=O > C-O > C–C > O–C=O. The root mean-square roughness measured by AFM analysis showed that the GO layer had a thickness of 1.1–1.3 nm (Figure 1c). To fabricate the GO/MS medium, the GO solution was sonicated for 2 h to maintain its homogeneity before use. The MS medium was added to the homogeneous GO solution and stirred to prevent aggregation (Figure 1d). The gelation of the agar powder added to the homogeneous GO/MS medium easily formed the solid agar medium. 

### 3.2. Influence of Graphene Oxide on the Plant Phenotype

**Figure 2:**
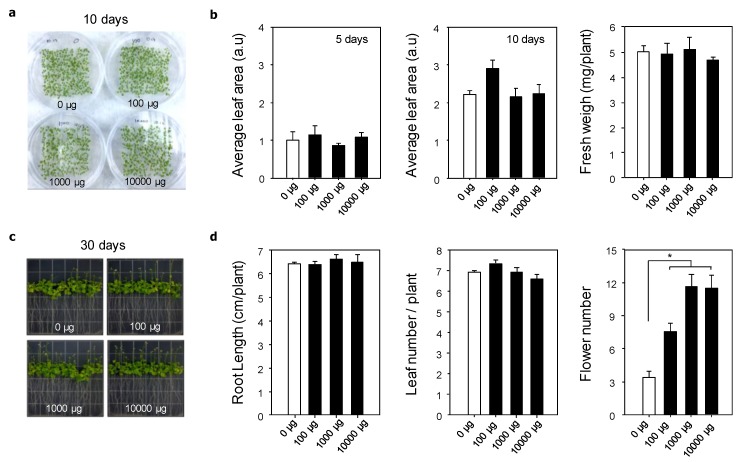
Effects of graphene oxide (GO) on the phenotype of *Arabidopsis thaliana* L. (**a**) Representative photograph of plant growth after 10 days of planting. (**b**) Effects of GO on the leaf area and fresh weight. (**c**) Representative photograph of plant growth after 30 days. (**d**) Effects of GO on the root length, leaf number, and flower number.

To confirm the influence of GO on the phenotype of *A. thaliana*, the leaf area, plant fresh weight, root length, leaf numbers, and flower numbers were evaluated after 5, 10, and 30 days of plant growth on the various GO/MS media (Figure 2). Figure 2b shows the leaf area after five and 10 days of planting. After five days, the leaf areas of *A. thaliana* grown on MS medium and on 1000 μg/L GO/MS medium were slightly greater than those grown on the 100 and 10,000 μg/L GO/MS media. The plant areas for all experimental groups were increased after 10 days of planting, where that grown on 1000 μg/L GO/MS medium was the highest. The areas of plants grown on the MS medium and 100 and 10,000 μg/L GO/MS media were not significantly different from one another. After 10 days of planting, the average fresh weight was 5.03 mg for the MS medium group, 4.98 mg for the 100 μg/L GO/MS medium group, 5.13 mg for the 1000 μg/L GO/MS medium group, and 4.50 mg for the 10,000 μg/L GO/MS medium group, with no significant differences among the different groups. These results indicated that a small amount of GO was not toxic to plants, suggesting the importance of the nanomaterial concentration for plant growth. 

**Figure 3:**
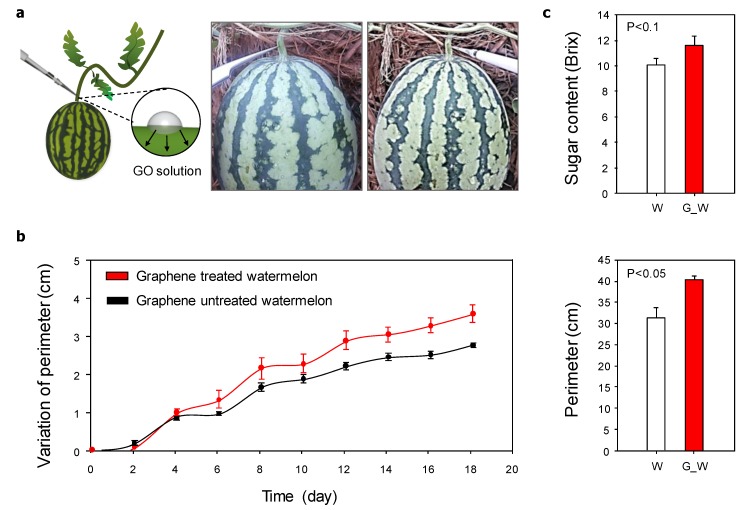
Effect of graphene oxide (GO) on watermelon. (**a**) Schematic of GO solution injection into the watermelon stem. (**b**) Effect of GO on the rate of perimeter change and the final watermelon perimeter. (**c**) Sugar contents in GO-treated watermelons (G_W) and untreated watermelons (W) (*n* = 4).

The root length is another important factor to consider when checking the cytotoxicity of nanomaterials on plant growth because the root adsorbs water and nutrients from the surroundings and has direct contact with the unique cues of GO, including its chemical, structural, and mechanical properties. After 30 days of planting, the average root length was 6.27 cm in the MS medium group, 6.23 cm in the 100 μg/L GO/MS medium group, 6.44 cm in the 1000 μg/L GO/MS medium group, and 6.31 cm in the 10,000 μg/L GO/MS medium group (Figure 3b). The photographs showed that GO was not aggregated on the root surface, and the branch roots had stretched into the surroundings. Begum et al. reported that graphene at 500, 1000, and 2000 mg/L was aggregated on the root surfaces, and the root length gradually decreased as the concentration of graphene was increased [18]. In our present study, the small amount of GO did not aggregate on the plant root surface and, therefore, it did not prevent the adsorption of water and nutrients, thus providing the proper stimulation to the roots. Next, we confirmed the leaf number after 30 days of planting because leaf production occurs between the 20th and 30th days [22]. The plant usually has 6–8 leaves by this period. However, the plants grown on 100 μg/L GO/MS medium had a slightly higher leaf number than those grown on the other media. In addition, the flower bud number was checked to confirm the growth differences between plants grown with or without GO. After the leaf production and rosette growth stages, *A. thaliana* begins the processes of inflorescence emergence and flowering to produce seeds [22]. Interestingly, the number of flower buds was significantly increased by GO (Figure 2d), where it was highest on plants grown on the 1000 and 10,000 μg/L GO/MS media (average of 11.67 and 11.33, respectively). The number of flower buds on the 100 μg/L GO/MS medium group (average of 7.67) was higher than that on the MS medium (average of 3.33). Although an in-depth study is still needed to reveal the effects of graphene on the plant genotype, the significant increases in the number of flower buds caused by the treatments of small amounts of GO suggest that this nanomaterial can be used as a platform to accelerate the stages of inflorescence emergence and flowering.

### 3.3. Direct Influence of Graphene Oxide on Watermelon

To confirm the direct influence of GO on crops, as a conceptual work, we used watermelon as a model crop and treated it with the fabricated nanomaterials. Each watermelon seedling was cultivated in a Styrofoam pot, and all stems of the plant except for the first stem were removed to improve the quality of the watermelon. We injected the GO solution directly into the stem of the watermelon fruit once a week for one month (Figure 3a). Since the relatively higher concentrations of GO had resulted in the highest number of flower buds (Figure 2), all watermelon experiments were conducted using the 10,000 μg/L GO solution. After injection of the GO solution, we compared the perimeter of the watermelon with that of the untreated watermelon once every two days. The watermelon perimeter increased gradually with time, but the rate of increase was faster for the GO-treated watermelons than for the untreated ones. Furthermore, the watermelon perimeter was significantly increased by the GO treatment, by an average of 5 cm over that of the untreated fruit. Additionally, it is commercially important for crops to have a high sugar content, and the average sugar content of watermelon is 8 Brix [23]. In this study, the Brix value of the GO-treated watermelon was 11.73 whereas it was 10.16 in the untreated watermelon, indicating that GO treatment had increased the sugar content of the fruit. On the basis of these results, it is suggested that GO affects the watermelon growth and quality positively, indicating the potential use of this nanomaterial as an agent for efficient crop cultivation. 

In this study, we investigated the use of small amounts of GO to provide positive effects on the phenotype and quality of plants and crops. To confirm the influence of GO, we fabricated the homogeneous GO/MS medium through a long period of sonication. Whereas the injection of GO solution into the MS medium caused the formation of aggregates, the injection of MS medium into the homogeneous GO solution resulted in the formation of a homogeneous and uniform GO/MS medium (data not shown). The model plant was cultured on this fabricated homogeneous GO/MS medium, and the small amounts of GO did not aggregate on the root surface. In previous studies, the root surface of various plants was covered by the nanomaterials, and subsequently their branch roots did not stretch into the surroundings [14,17]. Consequently, this prevented adsorption of water and nutrients onto the roots was stunted the overall growth of the plants. However, in our study, the plant phenotype on the GO/MS medium was similar overall to that on the MS medium, and GO was confirmed to be noncytotoxic to the plant. Stobiecka et al., revealed that GO was aggregated at the higher concentration than 80 µg/mL in a phosphate-buffered saline, and the stacking interactions of GO layer was accelerated at the higher concentration of >17 µg/mL in a phosphate buffer [24]. This means a possibility that the small amounts of GO used in this work might be minimized for the aggregation in the agar plate and the GO solution. In addition, the adsorption of water and nutrients would not have disturbed. Duan et al., demonstrated that high concentration of GO (>10 µg/mL) showed the promoted affinity to lipid membrane, and it might cause the formation of the pores on the cell membrane, which would be the main contributing factor of the cell death with the water permeation through the membrane [25]. Thus, it is very important to confirm the concentration of nanomaterials to prevent the aggregation and the pore formation for the improved cell viability. Begum and Fugetsu revealed about the cytotoxicity of GO with high concentration, which may accelerate the plant cell death [26]. The authors used a high concentration of < 80 mg/L on *A. thaliana* T87 cell suspension, and they confirmed the excessive accumulation of GO in the cell membrane and nuclei. This caused dysfunctions of the mitochondria, and ROS generation, which was the crux of the signaling pathway in cell death, including apoptosis and necrosis, was expedited. This happened in the plant cell culture with carbon nanotubes of high concentration [27]. The risks of GO with high concentrations are as follows: (1) the disturbance of the water and nutrient adsorption through the aggregation on the root surface; (2) the pore formation in the cell membrane with the high affinity; (3) mitochondrial dysfunction on the necrosis of plant cells; and (4) ROS generation, which may accelerate the cell death. To optimize the use of the nanomaterials on the plant growth, it would be very important to confirm the appropriate concentrations of nanomaterials to prevent the cell death for good cell viability.

The chronological progression of the principal growth stages of *A. thaliana* are germination, leaf production, rosette growth, inflorescence emergence, flowering, and silique ripening. To confirm the enhancement of plant growth, we checked the leaf area and fresh weight in the leaf production stage, and the root length, leaf number, and flower bud number in the inflorescence emergence and flowering stages. The leaf area, fresh weight, root length, and leaf number showed no significant differences between the GO/MS media and MS medium, but the flower bud number was noticeably increased by GO. Interestingly, the flower bud number increased gradually with increase in the GO concentration, with the 1000 and 10,000 μg/L GO/MS media giving similar results. Moreover, we observed the influence of the direct injection of the GO solution on crop growth and quality. The rate of change of the GO-injected watermelon perimeter was slightly higher than that of the untreated watermelon, and the overall perimeter of the GO-injected watermelon was also significantly higher. In particular, we confirmed the increased sugar content of the GO-injected watermelon relative to that of the untreated fruit. Pérez-de-Luque summarized the interaction of nanomaterials with plants [28]. Nanomaterials of 40–50 nm can penetrate plant tissues directly and affect plant germination and growth. In this study, we used a GO layer with a thickness of 1.1–1.3 nm, with dimensions ranging from a few nanometers to a few hundred nanometers generated through sonication for 2 h (Figure S1). Therefore, it was possible for the proposed GO to penetrate plant tissues and affect plant growth. To secure the evidence on the penetration and transportation of the GO in plants, we treated the rhodamine-loaded GO in the model plants (e.g., *Spinacia oleracea* L.). We measured the expression of a fluorescent signal, showing that the Rhodamine-loaded GO flakes were transported through the xylem upwards onto the stem. This revealed a possibility that the transported GO flakes might accumulate in the plant organs (Figure S2). In previous works, it has been reported that GO can penetrate the tissues and cells, which would control the functions of the living systems [18,29,30,31,32,33]. For example, Chen et al. conducted the translocation of various types of graphene using pea plants (*Pisum sativum* L.), and the bioaccumulation results of graphene shows that the GO of a 0.60 mg·mL^−1^ mainly accumulated in the root and stem after 10 days, and the accumulation in leaf slightly increased after 15 days [29]. The translocated GO into leaf inhibited the photosynthesis by distributing the oxygen evolving complex. Wang et al. demonstrated that the cellular entry of the GO using a simulation, showing the oxidized graphene sheet can pierce the lipid membrane of cell through the lipid change in the membrane [30]. In addition, Ratajczak et al. revealed that the GO conjugated with a supramolecular including adenosine-5′-triphosphate (ATP), FI-TC (fluorescein isothiocyanate), and survivin molecular beacon (SurMB) can be used as a carrier for gene delivery, and the GO usually interacted with the molecules through the hydrogen bonds’ formation and π-π stacking interactions [31,32]. Xie et al., reported the GO-based gene transcript levels of *Brassica napus* L. (*B. napus*) [34]. The authors treated the GO with difference concentrations (e.g., 0, 5, 25 mg/L) on *B. napus*. The transcript levels of ABA-related genes (ZEP, AAO, and NCED), IAA-related genes (ARF2, ARF8, IAA2, IAA3, IAA4, and IAA7), CTK-related genes (CKX1, CKX7, IPT2, IPT5, and IPT7), GA-related genes (SPY, RGA, GAMYB, GA3, and GA5), BAK1, CBP60, and calmodulin binding protein-like protein 1, the key genes for the phytohormone pathways and biosynthesis, were enhanced in the 5 mg/L GO-treated *B. napus*. Compared with the 5 mg/L GO-treated *B. napus*, the 25 mg/L GO-treated *B. napus* showed the reduction of the relative transcript levels that might influence the root length and adventitious root numbers. This work indicated that the treatment of the 5 mg/L GO was provided the positive effects for the phytohormone pathways and biosynthesis. In addition, Zhou’s groups tried to reveal the metabolic pathway-based mechanisms of the graphene family [35,36,37,38]. To confirm the metabolic regulation of the GO, the algal cells were exposed to the GO at 0.1, 1, and 10 mg/L. The algal cells had wrinkled structures after exposure of the GO, and it penetrated into the organelle of the agar cells. The authors suggested that the treatment of the GO affected on the increased accumulation of the unsaturated fatty acids and the inhibition of the sugars, and amino acids, which might cause the oxidative stress.

As mentioned above, the GO could be used for the plants and crops growth through the penetration and transportation, indicating that it is needed to address the problems on the human and animal health for future nano-agro-technology. Recently, Lahiani et al. confirmed the impact of the carbon-based nanomaterial contained tomato on the human epithelial cells extracted from lung metastasis of the colon carcinoma [39]. They treated the CNTs with a high concentration of 50 mg/L to tomato for 10 weeks, and the CNT-containing tomato with the concentration of 3 µg/mL was injected in the cell culture medium. Unlike the epithelial cells with the pristine CNT-based the cell culture medium, there was no major toxicity including gene expression levels in the CNT-containing tomato with the cell culture medium. The same group showed the potential of graphene as a food contact material using bioreactor-rotary culture systems [40]. The pristine graphene affected the intestinal microbiota, which might be the crucial factor in the equilibrium of the gastrointestinal tract, and the CFU of the aerobic bacteria was gradually decreased as the concentration of the pristine graphene increased. On the other hand, there were no effects on the CFU of the anaerobic bacteria. The authors mentioned that a higher concentration than 100 μg/mL graphene might show the side effects on the intestinal microbiome. These works provided information about the indirect effects of the nanomaterials on human and animal health, but it may be a first step towards the future nano-agro-technology. 

Although the GO has some potentials as a new tool to promote the plant and crop growth, there still remains many problems, including cytotoxicity. Hu et al. pointed out the knowledge gaps about the safety of the nanomaterials-based plant growth between the research and real fields [41]. For example, nanomaterials were exposed to plants with very short times without considering the life cycle of the used organisms. The organisms have complex replication processes, and the translocation of nanomaterials may cause the biological systems to gradually change their function over time [41,42]. Lazareva and Keller also estimated the release of engineered nanomaterials in the environmental media using the life cycle modeling approach, revealing that the distribution of the nanomaterials was strongly affected by the product consumption, wastewater treatment, and geographic locations [43]. Taken together, these reports suggest that the various conditions to treat nanomaterials, including GO, may be important issues for plant and crop growth. Although an in-depth study is still needed to reveal the direct and indirect effects of the nanomaterial-based plant and crop growth, we suggest that the nanomaterials with suitable concentrations would be a strategy to access the advanced plant and crop breeding science and engineering.

## 4. Conclusions 

In this study, we investigated the influence of small amounts of GO on a model plant and crop. We successfully fabricated a homogeneous GO/MS medium, and the phenotype of the model plant grown on the various GO/MS media was confirmed. GO of appropriate concentrations promoted *A. thaliana* growth and stability, indicated by the increases in the length of roots, the area of leaves, the number of leaves, and the formation of flower buds. In addition, the perimeter and sugar content of GO-injected watermelons were higher than those on the untreated fruits. We suggest that GO of appropriate concentrations may be used as an enabling strategy for plant and crop growth, and has great potential for use in plant and crop cultivation systems.

## Supplementary Materials

The following are available online at https://www.mdpi.com/2079-4991/10/4/758/s1, Figure S1: AFM images of the proposed GO. The proposed GO have a various size. Figure S2: Internalization of Rhodamine B-loaded GO. Bright-field microscope image of plant stem (left image) and fluorescence microscope image of plant stem (middle image) and Rhodamine B-loaded GO (middle images, white arrow) showing the uptake by plant xylem. Scale bar: 500 μm.
