# Supplementary material for: Graphene Oxide-Assisted Promotion of Plant Growth and Stability
Source: Nanomaterials (Basel). 2020 Apr 15;10(4):758. doi: 10.3390/nano10040758 (PMC7221628; doi:10.3390/nano10040758)
Supplement: Supplementary file 1 [file nanomaterials-10-00758-s001.pdf]

Supporting Information

Graphene Oxide-Assisted Promotion of Plant Growth and Stability

Sunho Park [1], Kyoung Soon Choi [2], Sujin Kim [1], Yonghyun Gwon [1] and Jangho Kim [1,*]

[1] Department of Rural and Biosystems Engineering, Chonnam National University, Gwangju 61186, Korea; preference9330@gmail.com (S.P.); kimsujin4172@gmail.com (S.K.); gyhjhj0716@gmail.com (Y.G.)

[2] Korea Basic Science Institute (KBSI), Daejeon 305-333, Korea; hanlalai83@gmail.com

* Correspondence: rain2000@jnu.ac.kr; Tel.: +82-062-530-5181

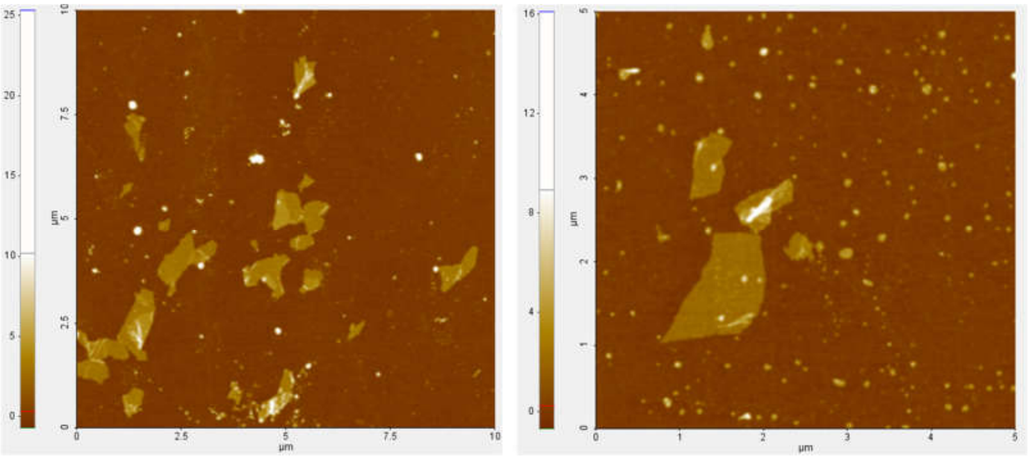

Figure S1. AFM images of the proposed GO. The proposed GO have a various size.

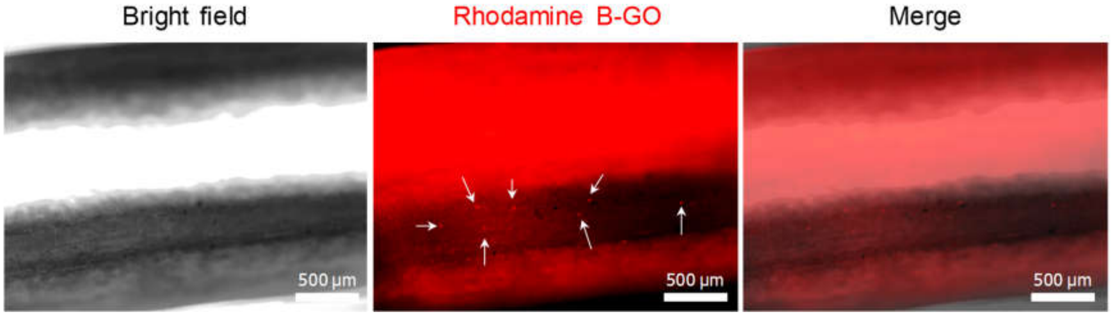

Figure S2. Internalization of Rhodamine B-loaded GO. Bright-field microscope image of plant stem (left image) and fluorescence microscope image of plant stem (middle image) and Rhodamine B-loaded GO (middle images, white arrow) showing the uptake by plant xylem. Scale bar: 500 μm.
